# Traumatic temporomandibular joint bony ankylosis in growing rats

**DOI:** 10.1186/s12903-022-02560-0

**Published:** 2022-12-09

**Authors:** Zhen Ma, Yiming Wang, Yang Xue, Wuyang Zhang, Dengke Li, Yuan Li, Guowei Li, Hongzhi Zhou, Xiangxiang Hu, Tiange Deng, Kaijin Hu

**Affiliations:** 1 State Key Laboratory of Military Stomatology, National Clinical Research and Center for Oral Diseases, Shaanxi Clinical Research Center for Oral Diseases and Department of Oral Surgery, School of Stomatology, The Fourth Military Medical University, 145 West Changle Road, Xi’an, 710032 China; 2 Division of Oral and Craniofacial Health Sciences, University of North Carolina Adams School of Dentistry, Chapel Hill, NC 27514 USA

**Keywords:** Temporomandibular joint bony ankylosis, Compound trauma, Joint space, Endochondral ossification, matrix metallopeptidase 13, Runt-related transcription factor 2

## Abstract

**Background:**

The pathogenesis of traumatic temporomandibular joint (TMJ) bony ankylosis remains unknown. This study aimed to explore the pathogenesis of traumatic TMJ bony ankylosis in a rat model.

**Methods:**

Twenty-four 3-week-old male Sprague–Dawley rats were used in this study. Excision of the whole disc, the fibrocartilage damage of the condyle and glenoid fossa, and narrowed joint space were performed in the left TMJ of the operation group to induce TMJ bony ankylosis (experimental side). The right TMJ underwent a sham operation (sham side). The control group did not undergo any operations. At 1, 4, and 8 weeks postoperatively, rats of the operation group were sacrificed and TMJ complexes were evaluated by gross observation, Micro-CT, histological examinations, and immunofluorescence microscopy. Total RNA of TMJ complexes in the operation group were analyzed using RNA-seq.

**Results:**

Gross observations revealed TMJ bony ankylosis on the experimental side. Micro-CT analysis demonstrated that compared to the sham side, the experimental side showed a larger volume of growth, and a considerable calcified bone callus formation in the narrowed joint space and on the rougher articular surfaces. Histological examinations indicated that endochondral ossification was observed on the experimental side, but not on the sham side. RNA-seq analysis and immunofluorescence revealed that Matrix metallopeptidase 13 (MMP13) and Runt-related transcription factor 2 (RUNX2) genes of endochondral ossification were significantly more downregulated on the experimental side than on the sham side. The primary pathways related to endochondral ossification were Parathyroid hormone synthesis, secretion and action, Relaxin signaling pathway, and IL-17 signaling pathway.

**Conclusions:**

The present study provided an innovative and reliable rat model of TMJ bony ankylosis by compound trauma and narrowed joint space. Furthermore, we demonstrated the downregulation of MMP13 and RUNX2 in the process of endochondral ossification in TMJ bony ankylosis.

## Background

Temporomandibular joint (TMJ) ankylosis is characterized by fibrous or bony adhesions after disease or trauma [1, 2], which affects physical and psychological health [3, 4]. Trauma is the most common cause of TMJ ankylosis [5–9], however the pathogenesis of traumatic TMJ bony ankylosis remains unclear. Prevention and treatment of traumatic TMJ bony ankylosis in clinical practice is therefore challenging, owing to the paucity of available information [10–12].

Conducting clinical trials of TMJ bony ankylosis in humans is limited by ethical considerations. Several animal models of TMJ bony ankylosis have been proposed over the last few decades [13–15]. Published studies describe injuries to the articular surfaces as procedures to induce TMJ ankylosis [15]. Large animal models are indispensable, as the size and anatomical structure of their TMJs are comparable to those of human beings [3]. Goats or sheep were the most common large animal models used in those studies [16]. Our previous study [17–19] revealed that compound trauma was critical for the establishment of TMJ bony ankylosis in goat or sheep models. However, commercially available biological and molecular assays for goats or sheep are currently limited owing to the lack of antibodies for these species [20–22], thus the pathogenesis and pathophysiology of TMJ bony ankylosis in large animal models are not possible to determine [22]. Therefore, TMJ bony ankylosis research on small animal models including rabbits [23–25], rats [15, 26], and mice [14, 27], are more conducive for molecular biology research. Traumatic TMJ bony ankylosis was stimulated using rabbits as small animal models, but the microstructure change of TMJ in rabbit models cannot be continuously and dynamically observed by Micro-CT [24]. Mice are too small to simulate traumatic TMJ bony ankylosis due to the high mortality rate. However, rats are cheap, fast, easy to house and implement in operations or studies, and antibodies for biological and molecular assays are available. Thus, rats are ideal animals for molecular biology research on traumatic TMJ bony ankylosis [28, 29]. Furthermore, TMJ bony ankylosis in rats has not previously been reported. Therefore, a small animal model of TMJ bony ankylosis based on Sprague–Dawley rats was proposed.

The TMJ cartilage is composed of dense layers of extracellular matrix (ECM) containing distributed chondrocytes [30]. Chondrocytes actively synthesize a large volume of ECM proteins such as hyaluronans, collagen, and proteoglycans [31, 32]. Yan et. al [33, 34] suggested that a series of Wnt signaling regulated bone formation and endochondral ossification during the formation of TMJ bony ankylosis, however, a few named genes were detected by real-time PCR. Our previous studies [17, 35] revealed that a large amount of ECM, chondrocytes, and endochondral ossification were observed in the joint space of TMJ bony ankylosis. Additionally, the gene expression pattern of sheep TMJ bony ankylosis was similar with the distraction osteogenesis (DO) process using gene chip analysis [35], however, only a few limited genes were found by gene chip technology. RNA-seq technology has led to the identification of more novel genes in small animal models than gene chip technology. Therefore, RNA-seq technology was superior to real-time PCR and gene chip technology for detecting genetic changes.

This study aims to 1) to establish a rat model of traumatic TMJ bony ankylosis in which articular disc removal, and damage to the fibrocartilage on the condyle and glenoid fossa, are combined with the narrowed joint space to verify whether the narrowed joint space and compound trauma lead to TMJ bony ankylosis; and 2) to systematically explore any alterations of genes and pathways in the expression of whole mRNA using RNA-seq technology in a rat model of traumatic TMJ bony ankylosis.

## Methods

### Ethics committee approval and animal care

Sprague–Dawley rats were provided by the Laboratory Animal Center of the Fourth Military Medical University (Xi'an, China) and all experiments were approved by the animal welfare ethics committee of the School of Stomatology [36] (Approval ID 2020–0950). The calculated sample size was 24 (PASS 11.0, Test for one correlation power analysis: Power = 0.9, α = 0.05, *R* = 0.74). Twenty-four male Sprague–Dawley rats at 3 weeks old (weight 60–80 g) were obtained from the Animal Center of the Fourth Military Medical University.

### Surgical procedures

**Fig. 1:**
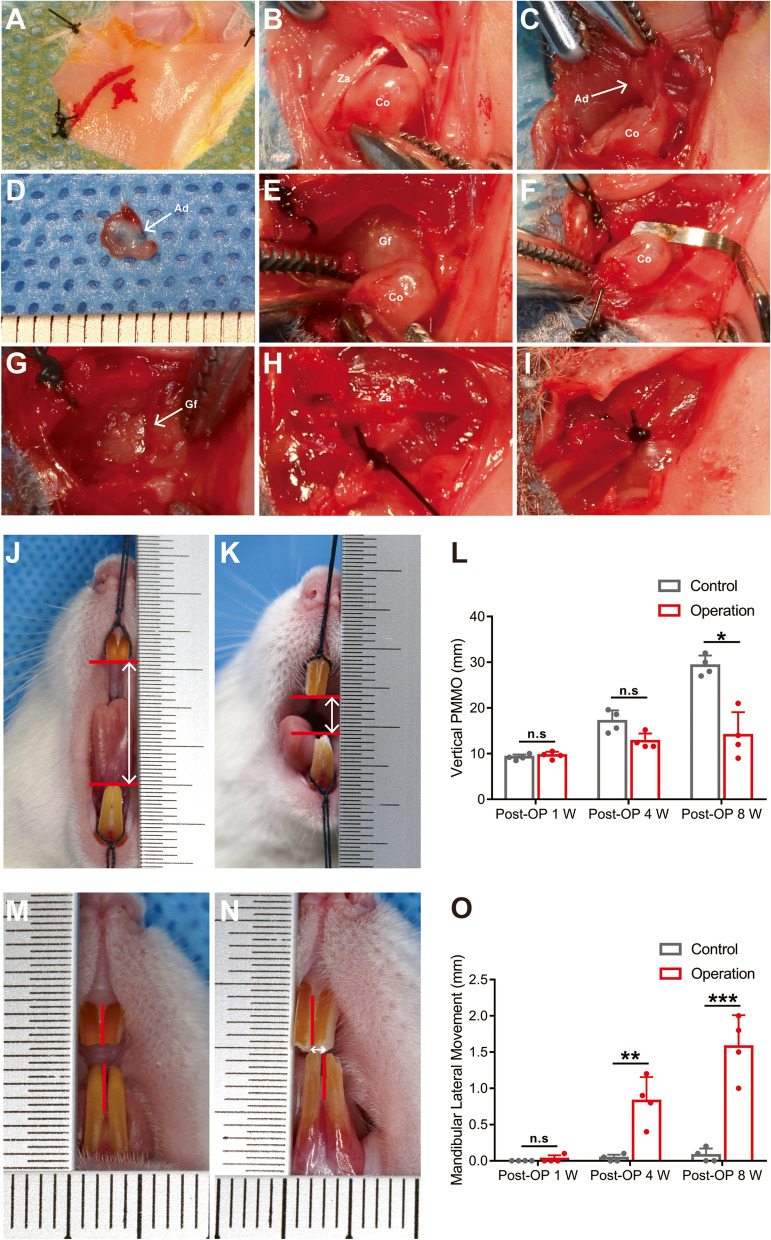
Surgical procedures on experimental side of operation group. **A** Label of preoperative incisions. **B** Exposure of the condyle. **C** Exposure of the articular disc (white arrow). **D** Resection of TMJ disc (white arrow). **E** Exposure of the joint space. **F** Damaging of the articular cartilage. **G** Carving grooves (white arrow) on the glenoid fossa. **H** Narrowing the joint space. **I** Suturing the articular capsule. **J** Measuring the distance between the maxillary and mandibular incisors as the vertical PMMO in control group (white vertical arrow). **K** Measuring the vertical PMMO in operation group (white vertical arrow). **L** Comparison of vertical PMMO between the two groups. **M** Measuring the distance between the middle line of the upper central incisors and the mandibular central incisors as mandibular lateral distance in control group (white horizontal arrow). **N** Measuring the mandibular lateral distance in operation group (white horizontal arrow). **O** Comparison of mandibular lateral distance between the two groups. *n* = 3. Data represents mean ± SD. *P* values, 2-tailed unpaired *t*-tests. Za, zygomatic arch; Co, condylar process; Ad, articular disc; Gf, glenoid fossa; Post-OP, post-operation; (*P < 0.05. **P < 0.01. ***P < 0.001)

Each Sprague–Dawley rat underwent general anesthesia with 2% isoflurane and oxygen. The TMJ region was isolated with sterile drapes. The temporal and preauricular regions were shaved and disinfected. In the operation group, the right TMJ underwent a sham operation (sham side) whereby a curved preauricular incision was made and the TMJ complex was exposed, then the wound was closed in layers. For the left TMJ of the operation group, the bony ankylosis-induced side (experimental side), a 1 cm long curved preauricular incision was made and the flap was lifted to expose the TMJ complex (Fig. 1A). The capsule was exposed by blunt dissection (Fig. 1B). The condyle was isolated with a periosteal elevator, and the articular disc was exposed by the separation of the lateral attachment of the disc through the joint space (Fig. 1C). The anterior and posterior attachments of the disc were then cut off and the articular disc was removed (Fig. 1D). The joint space was exposed by horizontal blunt dissection (Fig. 1E), the fibrocartilage on the condyle was removed using a Gracey scaler (Fig. 1F) and grid grooves were carved using an apex elevator on the surface of the glenoid fossa (Fig. 1G). The joint space was then narrowed (Fig. 1H), the capsule was sutured, and the wound was closed in layers (Fig. 1I). After surgery, each rat was administered an antibiotic (penicillin, 2 mg/100 g; X–Y Biotechnology) and an analgesic (pentazocine, 0.1 mg/100 g; X–Y Biotechnology) for 5 days. The control group did not undergo any operations.

The vertical passive maximum mouth opening (PMMO) and mandibular lateral movement were measured and recorded at 1, 4, and 8 weeks postoperatively according to our previous study [3]. Vertical PMMO was measured under general anesthesia as the distance between the maxillary and mandibular incisors upon the perpendicular application of 1.0 g of force to the jaw by a ruler scale (Fig. 1J, K). The force of 1.0 g was chosen based on pilot experiments which indicated that this amount of force was sufficient for the Sprague–Dawley rat jaws to reach the end-feel of ‘capsular feel’ or ‘springy-block’ [3]. Mandibular lateral movement was also measured and recorded under general anesthesia according to our previous study [18] (Fig. 1M, N).

### Sample preparation

All rats were euthanized with a lethal dose of pentobarbitone sodium by intraperitoneal injection at 1, 4, and 8 weeks postoperatively. All TMJ complexes were fixed in 4% paraformaldehyde. Then, TMJ complex samples were rinsed for 6 h with water and then decalcified in 17% EDTA for 6 weeks before embedding in paraffin. 4-μm sections were cut along the mesiodistal direction of the dentition.

### Micro-CT

All Sprague–Dawley rats underwent general anesthesia at 1, 4, and 8 weeks postoperatively. Their skulls were scanned by Micro-CT (Siemens AG) at a voltage of 80 kV, a current of 500 μA, and a resolution of 10 μm. Subsequently, three-dimensional (3D) images of TMJ complexes were reconstructed with the Inveon Research Workplace (Siemens AG). In the operation group, the region of interest (ROI) included three cubes (each 0.5 × 0.5 × 0.5 mm), which were selected along an imaginary line between the concave point of the condylar lateral neck and the upper edge of the zygomatic arch base. The morphological parameters of trabecular bone microarchitecture, including bone volume fraction (BV/TV), trabecular thickness (Tb.Th), bone specific surface (BS/BV), trabecular separation (Tb.Sp) and trabecular number (Tb.N), were measured.

### Histological examinations and immunofluorescence

The paraffin sections were stained with Hematoxylin and Eosin (H&E) and modified Safranine O-Fast Green FCF Cartilage Stain (Solarbio, G1371), according to the manufacturer's instructions. Slides were preincubated with 3% H_2_O_2_ for 10 min. Goat serum was used to block nonspecific binding, and then sections were incubated overnight at 4 °C with Coll II (1:1000, Abcam), MMP13 (1:250, Proteintech), RUNX2 (1:500, SANTA). Subsequently, sections were incubated with the corresponding secondary antibodies at room temperature for 1 h.

### RNA-seq analysis

Total RNA extracted from TMJ complexes was used for RNA sequencing by Beijing Genomics Institute (ShenZhen, China). The samples were sequenced at BGI (Cambridge, MA) with paired-end 100 bp in the BGISeq-500 platform. Each group had three replicates, and all sequence data were deposited in the Gene Expression Omnibus database. Pathway analysis was performed using the DAVID functional annotation bioinformatic tool.

### Statistical analysis

Data are presented as mean ± SD and were analyzed with SPSS 18.0 software (SPSS Inc, Chicago, IL, USA). The values of the control and experiment were compared at each time point. Micro-CT parametric data were compared by paired t-test. The vertical PMMO and the mandibular lateral movement between the control and operation groups were compared by independent t-test. P < 0.05 was considered statistically significant.

## Results

### Gross observation findings

All Sprague–Dawley rats could tolerate the compound trauma in the operation group, and no postoperative wound infection occurred. The vertical PMMO of the operation group was significantly lower than that of the control group at 4 and 8 weeks postoperatively (Fig. 1J, K). There was statistical significance in the vertical PMMO between the control and operation groups at 8 weeks postoperatively (Fig. 1L, *P* < 0.05). At 4 and 8 weeks postoperatively, the mandibular lateral movement of the operation group was significantly greater than that of the control group (Fig. 1M, N), and the difference was statistically significant between the two groups (Fig. 1O; Post-OP 4 W, *P* < 0.01; Post-OP 8 W, *P* < 0.001). At 8 weeks postoperatively, a clear limitation of vertical PMMO was observed in rats of the operation group.

**Fig. 2:**
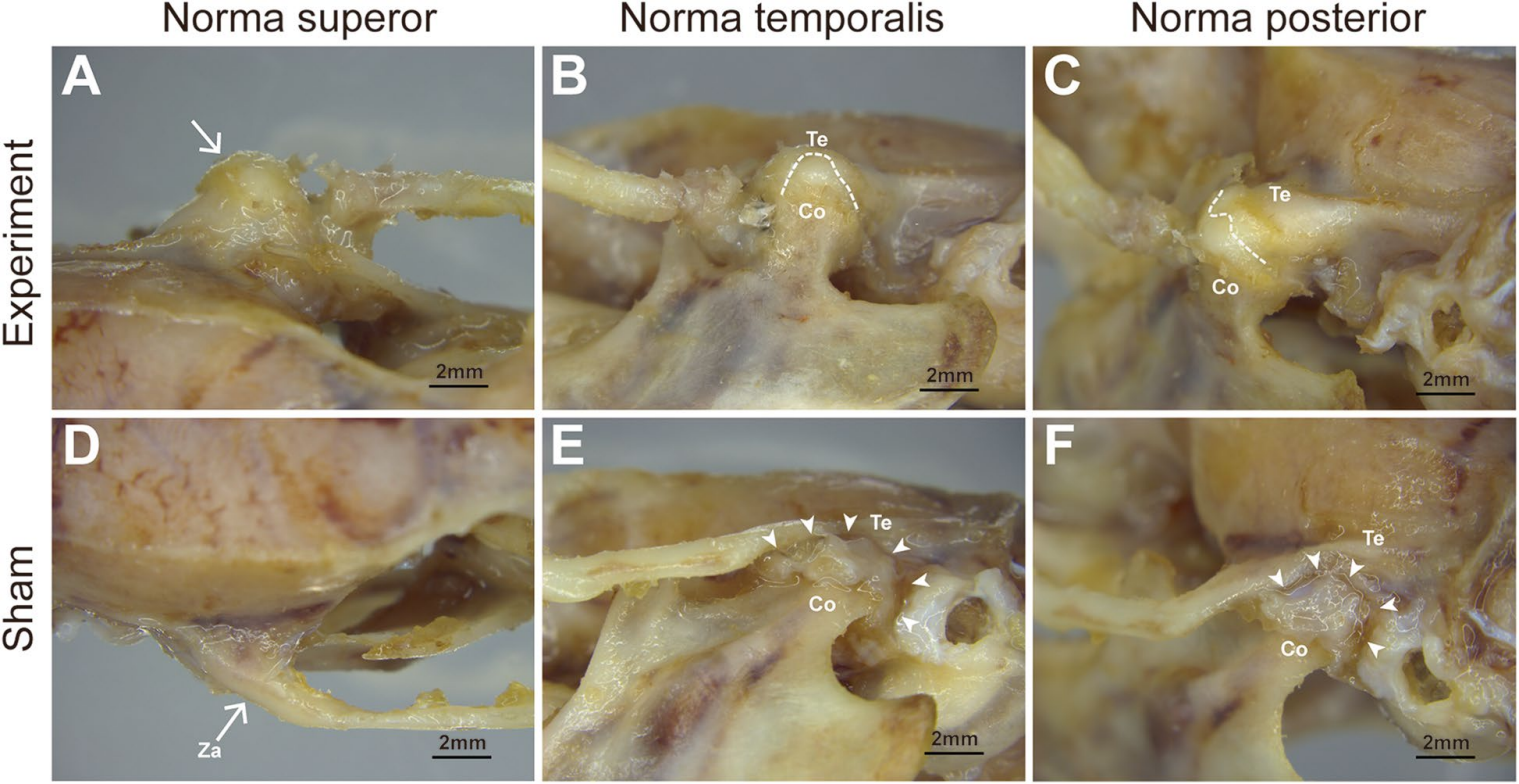
Gross observations of the TMJ complexes in experimental and sham side of operation group at 8 weeks postoperatively. **A** TMJ complexes of experimental side (white arrow). **B**, **C** Disappeared joint space and fusion line of condyle and temporal bone from different angles (white imaginary line). **D** Zygomatic arch of sham side (white arrow). **E**, **F** Joint space of sham side from different angles (white triangle arrow). Co: condylar process, Te, temporal bone, Za, zygomatic arch

On the experimental side, gross observations were made of the TMJ external appearance with a stereoscopic microscope at 8 weeks postoperatively (Fig. 2A-C). TMJ bony ankylosis and irregular new bone formation on the TMJ complex surfaces were observed (Fig. 2C). Moreover, bony adhesions were observed in the joint space of the experimental side (Fig. 2B, C; white dotted line).

On the sham side, bony ankylosis was absent (Fig. 2D-F). The glenoid fossa and articular cartilage did not exhibit fusion.

### Micro-CT findings

The microarchitectural data revealed that the image of TMJ bony ankylosis was observed on the experimental side but not on the sham side.

**Fig. 3:**
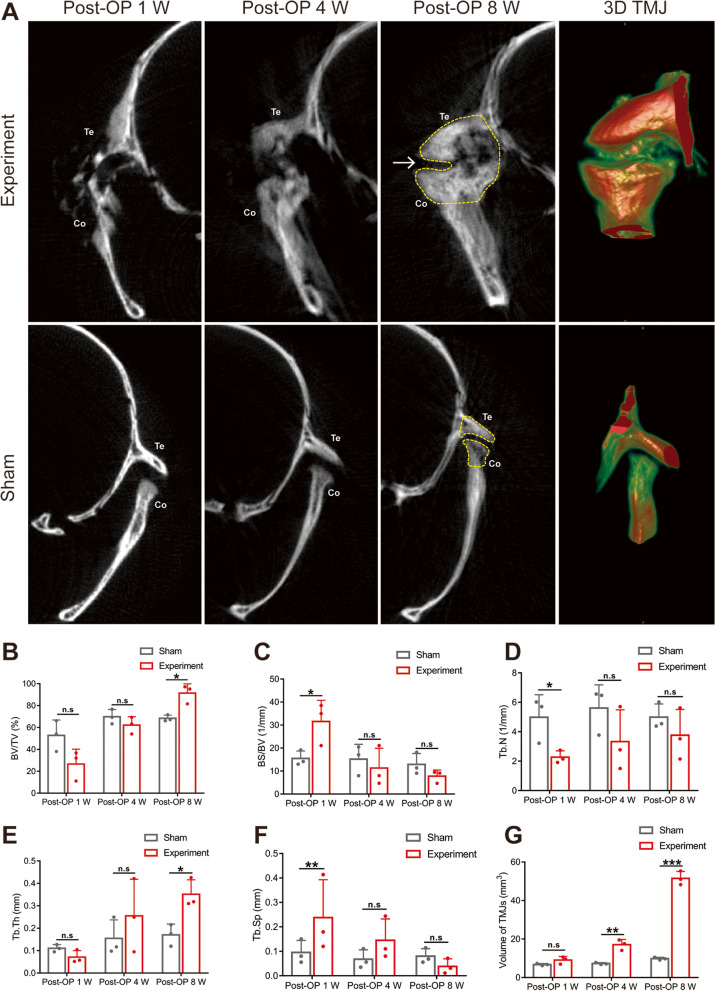
Micro-CT analysis of TMJ complexes on experimental side and sham side at 1, 4, and 8 weeks postoperatively. **A** The images of TMJ complexes along the coronal plane. Cartilaginous and bony fusion in the experimental side of the operation group at 8 weeks postoperatively (white arrow). **B-G** BV/TV (%), BS/BV (1/mm), Tb/N (1/mm), Tb.Th (mm), Tb.Sp (mm) and the volume of TMJ complexes were analyzed. Data represent mean ± SD. *P* values, 2-tailed unpaired *t*-tests; Post-OP, post-operation; (*P < 0.05. **P < 0.01. ***P < 0.001)

On the experimental side, some new cartilage and bone were observed at 1 week postoperatively. There was moderate new cartilage and bone on the experimental side at 4 weeks postoperatively and joint space was narrower at 4 weeks than that at 1 week postoperatively. At 8 weeks postoperatively, TMJ bony ankylosis was found on the experimental side, whereby the joint space had nearly disappeared and was filled with abundant new cartilage and bone (Fig. 3A; white arrow) and the degree of calcification increased over time. The morphological parameters of the trabecular bone microarchitecture were calculated (Fig. 3B-F). The volume of TMJ complexes was 51.54 ± 3.584 mm^3^ at 8 weeks postoperatively (Fig. 3G).

**Fig. 4:**
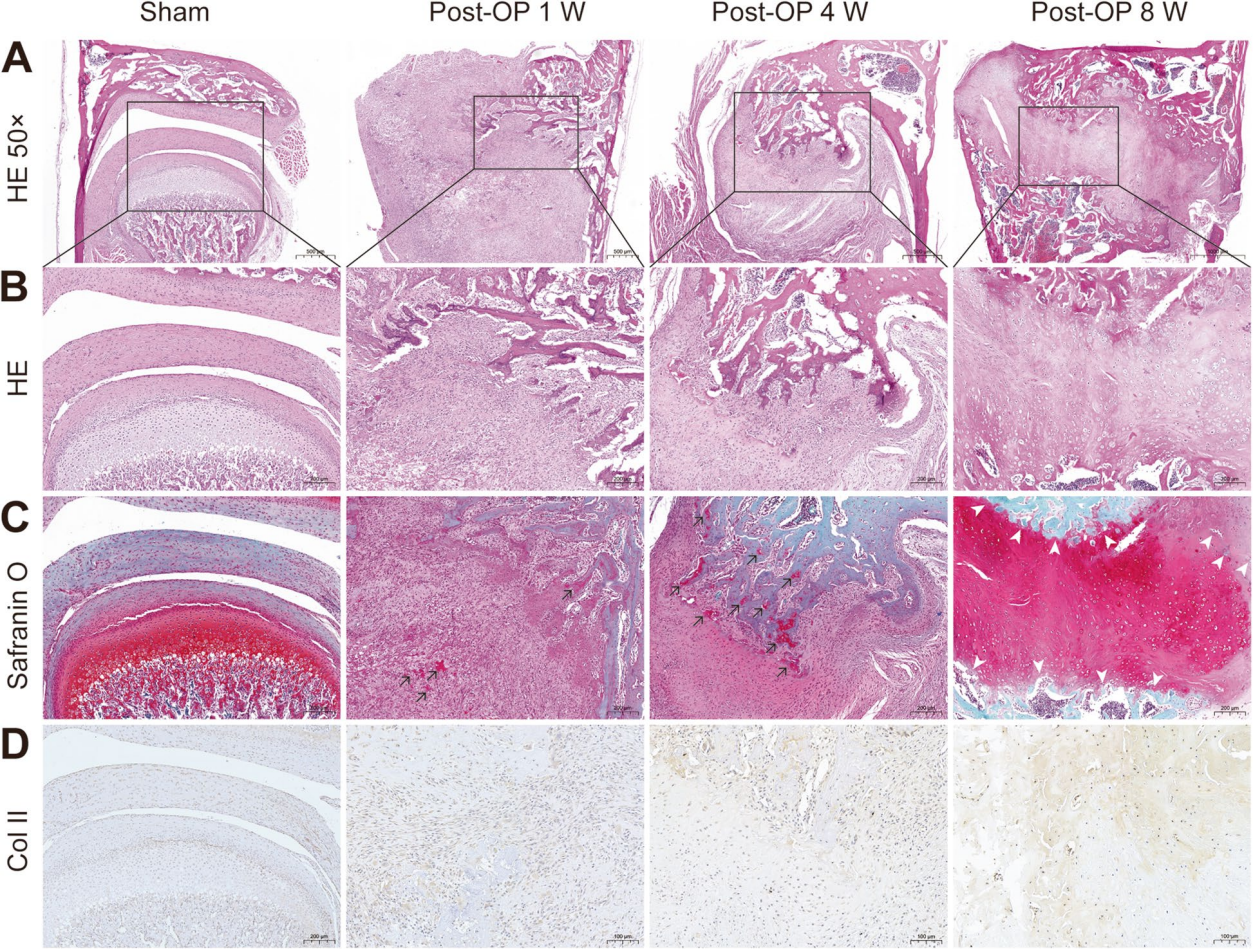
Histological sections of TMJ complexes on the experimental side and sham side (coronal section analysis). **A**, **B** Representative images of H&E staining paraffin sections. **C** Representative images of Safranin O staining. **D** Col II immunohistochemical staining of cartilage

On the sham side, TMJ regular upper and lower articular surfaces were observed, however TMJ bony ankylosis was absent. There was no new cartilage or bone in the joint space of the sham side at 1, 4, and 8 weeks postoperatively. The image of the joint space showed that it was slightly narrowed. Similarly, the morphological parameters of the trabecular bone microarchitecture were calculated. (Fig. 4B-F). The volume of TMJ complexes was 9.827 ± 0.716 mm^3^ at 8 weeks postoperatively (Fig. 4G).

BV/TV (*P* = 0.014) and Tb.Th (*P* = 0.172) of the experimental side TMJ complexes were significantly increased than that on the sham side at 8 weeks postoperatively (Fig. 3B, E). BS/BV (*P* = 0.033) and Tb.Sp (*P* = 0.258) of the experimental side TMJ complexes increased more at 1 week postoperatively than they did on the sham side (Fig. 3C, F). Tb.N (*P* = 0.811) of the experimental side was lower than that on the sham side at 1 week postoperatively (Fig. 3D). The volume difference of TMJ complexes was statistically significant between the experimental and sham sides at 4 and 8 weeks postoperatively (Fig. 3G) (Post-OP 4 W, *P* = 0.004; Post-OP 8 W, *P* = 0.000), There was no significant difference between the other parameters between the two sides at other times.

### Histological examination

All TMJ complexes on the experimental and sham sides were verified histologically (Fig. 4A-D).

On the experimental side, H&E staining showed a small amount of new cartilaginous matrix and cartilage cells, and a large amount of fibrous tissue in the joint space at 1 and 4 weeks postoperatively (Fig. 4A). Bony ankylosis was observed in the joint space, which was abundantly filled with new cartilaginous matrix, cartilage cells, endochondral ossification, and a small amount of fibrous tissue at 8 weeks postoperatively (Fig. 4A, B). Safranin O staining was used to evaluate the amount of red glycosaminoglycan (GAG) in the cartilage of TMJ complexes (Fig. 4C). A small amount of GAG was observed in the joint space at 1 and 4 weeks postoperatively (Fig. 4 C; black arrow), however GAG was abundant and distributed irregularly in the joint space at 8 weeks postoperatively, when endochondral ossification and cartilage margin ossification proceeded simultaneously (Fig. 4C; white arrow). IHC staining for Col II showed that the Col II-positive cells contributed 16.5%, 34.2%, and 44.3% of all Col II cells at 1, 4, and 8 weeks after the operation, respectively.

On the sham side, bony ankylosis and endochondral ossification were absent in the joint space, there was no statistical significance in GAG and Col II expression at 1, 4, and 8 weeks postoperatively.

### RNA-seq analysis

**Fig. 5:**
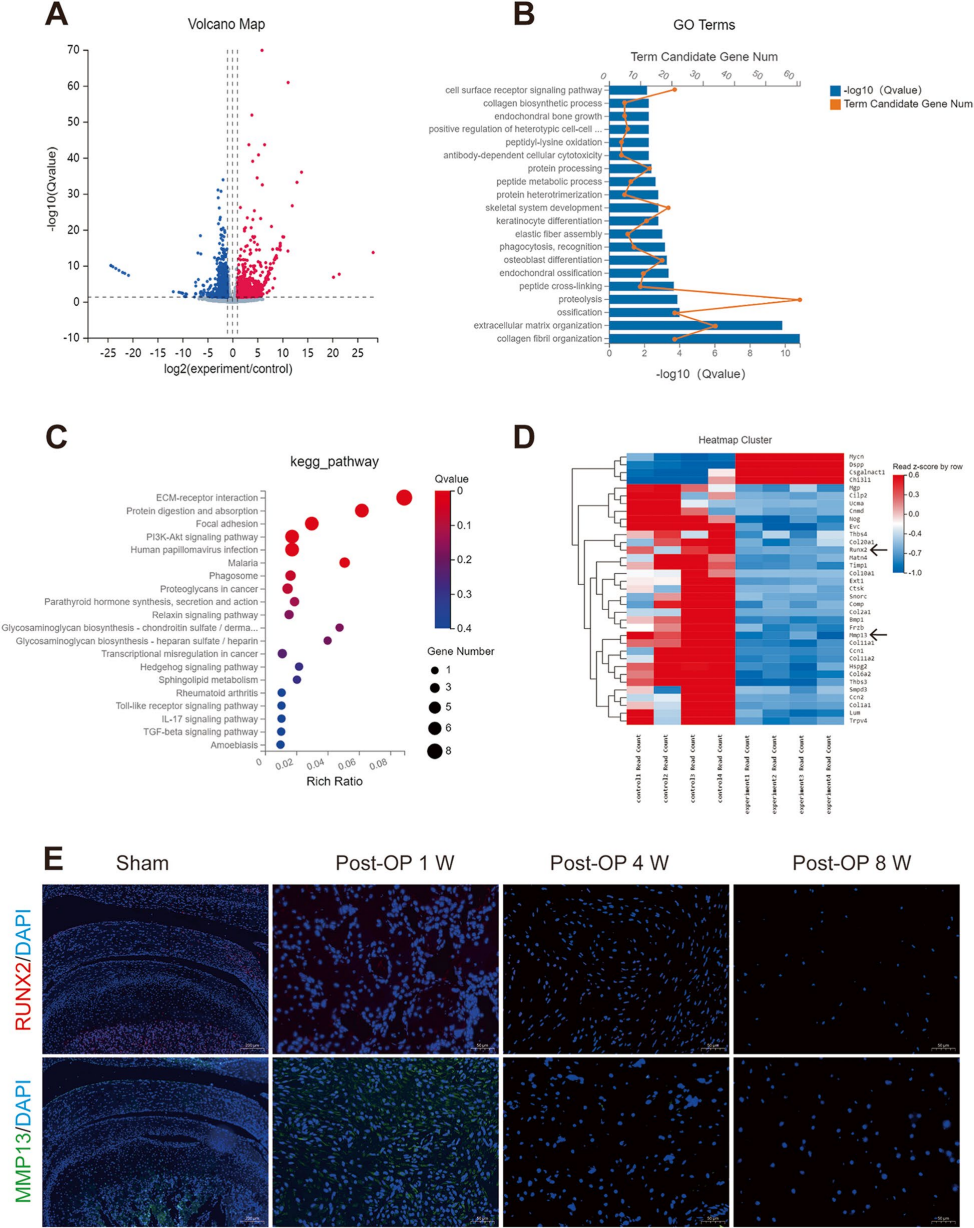
RNA-seq analysis and the immunofluorescence of RUNX2 and MMP13 genes with TMJ complexes between experimental side and sham side. **A** Volcano plot demonstrating genes differentially upregulated and downregulated. The biological coefficient of variation plot indicates biological variation within experimental side and sham side to be between 0.4 and 0.5. **B** Significant enriched GO terms of TMJ complexes between experimental side and sham side. **C** KEGG pathway terms of differentially expressed genes. **D** Heatmap of the differentially expressed genes between experimental side and sham side. **E** The immunofluorescence of RUNX2 and MMP13 genes between experimental side and sham side

RNA-sequencing was performed for dissected TMJ complexes on both the experimental side and sham side. Whole TMJ-complex RNA-Seq analysis identified transcripts corresponding to 37,246 genes. A volcano plot identified both upregulated and downregulated genes, while the biological coefficient of variation was between 0.4 and 0.5, within an acceptable range variation (Fig. 5A). The x-axis is the log2 scale of the fold change of gene expression between the two sides (log2 (fold change)). Negative/positive values indicate downregulation/upregulation. The y-axis is the minus log10 scale of the adjusted *p* values (-log10), which indicates the significant differences in the level of expression. The red dots represent significantly upregulated genes with at least a twofold change, while the blue dots represent significantly downregulated genes with at least a twofold change (Fig. 5A).

At 8 weeks postoperatively, differentially expressed genes (DEGs) were enriched in 101 terms, including 56 biological process (BP) terms, 16 cellular component (CC)terms and 29 molecular function (MF) terms. DEGs with endochondral ossification primarily included MMP13 and RUNX2. MMP13-related terms were as followed: in the BP domain, the meaningful enriched GO terms focused on extracellular matrix organization (GO:0,030,198), ossification (GO:0001503), proteolysis (GO:0006508), endochondral ossification (GO:0001958), and osteoblast differentiation (GO:0001649) (Fig. 5B). The most enriched CC terms were correlated with the extracellular space (GO:0,005,615). The MF terms were closely related to the extracellular matrix structural constituent (GO:0005201), and platelet-derived growth factor binding (GO:0048407). RUNX2-related terms were as followed: in the BP domain, the meaningful enriched GO terms focused on endochondral ossification (GO:0001958), ossification (GO:0001503), osteoblast differentiation (GO:0001649), protein processing (GO:0016485), and skeletal system development (GO:0001501) (Fig. 5B). RUNX2 was not enriched in the CC or MF terms. Kyoto Encyclopedia of Genes and Genomes (KEGG) analysis of the 101 DEGs identified 20 pathways, which are shown a bubble diagram. The KEGG pathways with endochondral ossification that were enriched at 8 weeks after the operation primarily included Parathyroid hormone synthesis, secretion and action, the Relaxin signaling pathway, and the IL-17 signaling pathway (Fig. 5C). We used a heatmap to show the distribution of DEGs between the experimental and sham sides. Red and blue stripes represent genes with high or low expressions, respectively (Fig. 5D). MMP13 and RUNX2 showed a significant difference between the experimental and sham sides (Fig. 5D; black arrow).

#### Immunofluorescence staining

Immunofluorescence staining demonstrated significantly lower expression of MMP13 and RUNX2 in the experimental side compared to the sham side at different times postoperatively (Fig. 5E). The RUNX2-positive cells were 38.9%, 16.2%, and 3.9%, and the MMP13-positive cells were 46.3%, 27.5%, and 5.3% at 1, 4, and 8 weeks postoperatively.

## Discussion

The present study showed that a new rat model of traumatic TMJ bony ankylosis was successfully established, and the genes and pathways related endochondral ossification were initially identified. Limitation of mouth opening and gross observation were found to be critical diagnostic indexes of TMJ bony ankylosis [3, 11, 17]. At 8 weeks postoperatively, the lower vertical PMMO and greater mandibular lateral movement of the operation group were significantly higher than those of the control group. Moreover, bony adhesions and irregular new bone formation on the TMJ complex surfaces were observed by gross observation. Therefore, the established Sprague–Dawley rat model for TMJ bony ankylosis was reliable in the present study. So far, traumatic TMJ ankylosis has been associated with several risk factors, including some types of condylar fractures, disc rupture/displacement, TMJ damage, and prolonged immobilization of the mandible [17]. Our previous study showed that TMJ bony ankylosis did not occur with minor compound trauma of the TMJ complexes [19]. Yan et al. [37] revealed that TMJ bony ankylosis was successfully established using severe compound trauma in infant sheep. Further, our recent study revealed that traumatic TMJ bony ankylosis was also associated with severe compound trauma, including disc displacement or rupture, and condylar and glenoid fossa damage [3, 17]. All of the above-mentioned studies indicate that severe compound trauma is necessary for the development of TMJ bony ankylosis.

Based on clinical observations and animal experiments, our previous studies [11, 17] and those of Zhang et al. [24, 33] found that TMJ bony ankylosis occurred during the growth and development phases of infancy and childhood. A previous review reported that narrowing the joint space by closing contact of the two injured articular surfaces, could induce TMJ bony ankylosis in young people [38]. Our recent study revealed that a narrowed joint space was reflected by the decreased vertical height of the mandibular ramus in a young sheep [17]. Cheung et al. [39] also reported that a narrowed joint space using bone grafts in the TMJ between the two injured articular surfaces could induce TMJ bony ankylosis in young sheep. Further, Li et al. [40] showed that TMJ bony ankylosis could not occur without a narrowed joint space in growing rats. The above-mentioned studies all support that a narrowed joint space and TMJ compound trauma in infancy are necessary for TMJ bony ankylosis. Recently, our pilot experiments revealed that the adult rat model of traumatic TMJ bony ankylosis could not be successfully established by severe compound trauma without a narrowed joint space. However, in the present study, for the first time, we successfully established a rat animal model of traumatic TMJ bony ankylosis using severe compound trauma and a narrowed joint space in infant rats. Therefore, the present study indicated that severe TMJ compound trauma (including disc displacement or rupture, and condylar and glenoid fossa damage) and a narrowed joint space during infancy were necessary for TMJ bony ankylosis. More importantly, joint space narrowing will inevitably occur in the process of TMJ bony ankylosis.

High spatial resolution of Micro-CT is a critical technical advancement that has allowed researchers to monitor the progression of bone disease in small animal models [41]. Micro-CT images revealed that low, moderate, and abundant levels of new cartilage and bone were observed in the joint space of the experimental side at 1, 4, and 8 weeks postoperatively. The results demonstrated that traumatic TMJ bony ankylosis worsened over time on the experimental side, which led to further limitation of mouth opening, and these findings were consistent with our previous studies [3, 17]. Moreover, according to 3D Micro-CT images, there was a larger volume and narrower joint space of TMJ complexes over time on the experimental side compared to that on the sham side. Micro-CT images also showed that there was more new bone on the experimental side than on the sham side according to a decrease in BS/BV, and an increase in BV/TV. These findings showed that new bone gradually grew over time on the experimental side, which revealed that TMJ bone ankylosis and limited mouth opening became more severe. Additionally, Micro-CT findings revealed the degree of calcification of the bony fusion area, which was visualized as vague radiolucent zones, lower than that of cortical bone but higher than that of the residual joint space. These results may explain why mouth opening affects the degree of calcification between the two injured articular surfaces, which further explains why none of the rats exhibited trismus in the present study. These findings were also consistent with our previous study [3, 17].

H&E staining, Safranin O staining, and IHC staining for Col II showed that a new cartilaginous matrix, cartilage cells, and endochondral ossification were abundant in the joint space of the experimental side, but not in the joint space of sham side, which also indicated that the bony ankylosis was formed in the experimental side. Moreover, new cartilaginous matrix, cartilage cells, and endochondral ossification gradually grew over time in the joint space of the experimental side, which further explained that the joint space became gradually narrower and the severity of bony ankylosis was more obvious over time. The expression of Col II on the experimental side was much higher than that of the sham side at 1, 4, and 8 weeks postoperatively. Col II-labeled new cartilaginous matrix was increased in the joint space of the experimental side at 1, 4, and 8 weeks postoperatively. The results revealed that the severity of bony ankylosis and limitation of mouth opening became more severe, which was in line with our previous studies [3, 17]. Our previous study showed that collagen type II was visible in the early healing process of the condylar fracture which blocked the functions of lateral pterygoid muscles. This was more prone to endochondral ossification because collagen type II is specifically produced by chondrocytes, which are the main organic component of a cartilage matrix [42]. Although fibro-osseous ankylosis was developed in a sheep model by Yan et. al [37] by Safranin O staining, IHC staining with Col II was absent. However, the present results proved that endochondral ossification correlated with new bone formation in the TMJ bony ankylosis using Sprague–Dawley rats.

RNA-seq analysis showed that MMP13 and RUNX2 were closely related to the pathways of Parathyroid hormone synthesis, and secretion and action, according to the GO terms of endochondral ossification. MMP13 and RUNX2 are involved in bone remodeling and early stages of endochondral bone formation [43]. MMP13 has been proven to be a downstream target of RUNX2 [44] which directly regulates MMP13 promotor activity and MMP13 expression in vivo [45]. Takeuchi et. al [46] reported that cartilage-specific overexpression of MMP13 induces cartilage degeneration in precocious arthritis. However, immunofluorescence staining demonstrated that expression of MMP13 and RUNX2 were lower on the experimental side than they were on the sham side. The present study found that the decreased MMP13 expression was accompanied by an increased expression of type II collagen, and we deduced that it was this decrease of MMP13 that led to the increased expression of type II collagen because MMP13 was reported to be involved in degrading type II collagen [47, 48]. Therefore, the expression of MMP13 was negatively correlated with the number of chondrocytes, cartilage ECM, and endochondral bone formation in the process of TMJ bony ankylosis. Similarly, the present study found that the expression of RUNX2 was negatively correlated with the number of chondrocytes, cartilage ECM, and endochondral bone formation in the process of TMJ bony ankylosis, which was in line with previous studies [49, 50]. Thus, in future studies we will further explore the roles of MMP13 and RUNX2 in the pathogenesis of endochondral ossification of traumatic TMJ bony ankylosis by focusing on the Parathyroid hormone synthesis and secretion and action pathways.

This study had some limitations, one of which was the short time period of postoperative observations, therefore our subsequent experiments will extend the observation time to explore the characteristics of cartilage bone in TMJ bony ankylosis. Moreover, the total RNA of the operation group 4 weeks after the operation could not be extracted for RNA-seq analysis due to some uncontrollable factors.

## Conclusions

In conclusion, the present study successfully provided a novel and reliable small animal model for future studies of traumatic TMJ bony ankylosis. Moreover, the pathogenesis of traumatic TMJ bony ankylosis was preliminarily explored and it was found that MMP13 and RUNX2 contribute to endochondral ossification. These findings will provide new therapeutic strategies targeting RUNX2 and MMP13 to alleviate TMJ bony ankylosis in the future.

## Data Availability

The datasets used and/or analyzed during the current study are available from the corresponding author on reasonable request.

## References

[1] Acri TM, Shin K, Seol D, Laird NZ, Song I, Geary SM, Chakka JL, Martin JA, Salem AK (2019). Tissue Engineering for the Temporomandibular Joint. Adv Healthc Mater.

[2] Sawhney CP (1986). Bony ankylosis of the temporomandibular joint: follow-up of 70 patients treated with arthroplasty and acrylic spacer interposition. Plast Reconstr Surg.

[3] Deng TG, Liu CK, Wu LG, Liu P, Wang JJ, Sun XZ, Zhang LL, Ma Y, Chen CS, Ding YX (2020). Association between maximum mouth opening and area of bony fusion in simulated temporomandibular joint bony ankylosis. Int J Oral Maxillofac Surg.

[4] Chouinard A-F, Kaban LB, Peacock ZS (2018). Acquired Abnormalities of the Temporomandibular Joint. Oral Maxillofac Surg Clin North Am.

[5] Chidzonga MM (1999). Temporomandibular joint ankylosis: review of thirty-two cases. Br J Oral Maxillofac Surg.

[6] el-Sheikh MM (1999). Temporomandibular joint ankylosis: the Egyptian experience. Ann R Coll Surg Engl.

[7] Zhang Y, He DM, Ma XC (2006). Posttraumatic temporomandibular joint ankylosis: clinical development and surgical management. Zhonghua Kou Qiang Yi Xue Za Zhi.

[8] Long X, Li X, Cheng Y, Yang X, Qin L, Qiao Y, Deng M (2005). Preservation of disc for treatment of traumatic temporomandibular joint ankylosis. J Oral Maxillofac Surg.

[9] Zhang PP, Liang SX, Wang HL, Yang K, Nie SC, Zhang TM, Tian YY, Xu ZY, Chen W, Yan YB (2021). Differences in the biological properties of mesenchymal stromal cells from traumatic temporomandibular joint fibrous and bony ankylosis: a comparative study. Anim Cells Syst.

[10] Haq J, Patel N, Weimer K, Matthews NS (2014). Single stage treatment of ankylosis of the temporomandibular joint using patient-specific total joint replacement and virtual surgical planning. Br J Oral Maxillofac Surg.

[11] Liu CK, Meng FW, Tan XY, Xu J, Liu HW, Liu SX, Huang HT, Yan RZ, Hu M, Hu KJ (2014). Clinical and radiological outcomes after treatment of sagittal fracture of mandibular condyle (SFMC) by using occlusal splint in children. Br J Oral Maxillofac Surg.

[12] Li JM, An JG, Wang X, Yan YB, Xiao E, He Y, Zhang Y (2014). Imaging and histologic features of traumatic temporomandibular joint ankylosis. Oral Surg Oral Med Oral Pathol Oral Radiol.

[13] Wang HL, Zhang PP, Meng L, Liang SX, Liu H, Yan YB (2018). Preserving the Fibrous Layer of the Mandibular Condyle Reduces the Risk of Ankylosis in a Sheep Model of Intracapsular Condylar Fracture. J Oral Maxillofac Surg.

[14] Ouyang N, Zhu X, Li H, Lin Y, Shi J, Dai J, Shen G (2018). Effects of a single condylar neck fracture without condylar cartilage injury on traumatic heterotopic ossification around the temporomandibular joint in mice. Oral Surg Oral Med Oral Pathol Oral Radiol.

[15] Porto GG, Vasconcelos BC, Fraga SN, Castro CM, Andrade ES (2011). Development of temporomandibular joint ankylosis in rats using stem cells and bone graft. Int J Oral Maxillofac Surg.

[16] Almarza AJ, Brown BN, Arzi B, Ângelo DF, Chung W, Badylak SF, Detamore M (2018). Preclinical Animal Models for Temporomandibular Joint Tissue Engineering. Tissue Eng Part B Rev.

[17] Deng TG, Liu CK, Liu P, Zhang LL, Wu LG, Zhou HZ, Ding YX, Hu KJ (2016). Influence of the lateral pterygoid muscle on traumatic temporomandibular joint bony ankylosis. BMC Oral Health.

[18] Meng F, Hu K, Kong L, Zhao Y, Liu Y, Zhou S (2010). Veterinary and radiological evaluations of open and closed treatment of type B diacapitular (intracapsular) fractures of the mandibular condyle in sheep. Br J Oral Maxillofac Surg.

[19] Liu CK, Liu P, Meng FW, Deng BL, Xue Y, Mao TQ, Hu KJ (2012). The role of the lateral pterygoid muscle in the sagittal fracture of mandibular condyle (SFMC) healing process. Br J Oral Maxillofac Surg.

[20] Sassu EL, Kangethe RT, Settypalli TBK, Chibssa TR, Cattoli G, Wijewardana V (2020). Development and evaluation of a real-time PCR panel for the detection of 20 immune markers in cattle and sheep. Vet Immunol Immunopathol.

[21] Acevedo Jiménez GE, Tórtora Pérez JL, Rodríguez Murillo C, Arellano Reynoso B, RamírezÁlvarez H (2021). Serotyping versus genotyping in infected sheep and goats with small ruminant lentiviruses. Vet Microbiol.

[22] Monteiro J, Guastaldi FPS, Troulis MJ, McCain JP, Vasconcelos B (2021). Induction, Treatment, and Prevention of Temporomandibular Joint Ankylosis-A Systematic Review of Comparative Animal Studies. J Oral Maxillofac Surg.

[23] Tuncel U, Kostakoglu N, Turan A, Markoç F, Gokçe E, Erkorkmaz U (2014). The use of temporalis muscle graft, fresh and cryopreserved amniotic membrane in preventing temporomandibular joint ankylosis after discectomy in rabbits. J Craniomaxillofac Surg.

[24] Zhao L, Xiao E, He L, Duan D, He Y, Chen S, Zhang Y, Gan Y (2020). Reducing macrophage numbers alleviates temporomandibular joint ankylosis. Cell Tissue Res.

[25] Tuncel U, Ozgenel GY (2011). Use of human amniotic membrane as an interpositional material in treatment of temporomandibular joint ankylosis. J Oral Maxillofac Surg.

[26] Rodrigues L, Corrêa L, Luz JG (2011). Healing of displaced condylar process fracture in rats submitted to protein undernutrition. J Craniomaxillofac Surg.

[27] Dai J, Ouyang N, Zhu X, Huang L, Shen G (2016). Injured condylar cartilage leads to traumatic temporomandibular joint ankylosis. J Craniomaxillofac Surg.

[28] da Silva MA, Oliveira JM, Reis RL (2018). Small Animal Models. Adv Exp Med Biol.

[29] Dias IR, Viegas CA, Carvalho PP (2018). Large Animal Models for Osteochondral Regeneration. Adv Exp Med Biol.

[30] Li B, Guan G, Mei L, Jiao K, Li H (2021). Pathological mechanism of chondrocytes and the surrounding environment during osteoarthritis of temporomandibular joint. J Cell Mol Med.

[31] Akkiraju H, Nohe A (2015). Role of Chondrocytes in Cartilage Formation, Progression of Osteoarthritis and Cartilage Regeneration. J Dev Biol.

[32] Goldring MB (2012). Chondrogenesis, chondrocyte differentiation, and articular cartilage metabolism in health and osteoarthritis. Ther Adv Musculoskelet Dis.

[33] Yan YB, Li JM, Xiao E, An JG, Gan YH, Zhang Y (2014). A pilot trial on the molecular pathophysiology of traumatic temporomandibular joint bony ankylosis in a sheep model. Part I: Expression of Wnt signaling. J Craniomaxillofac Surg.

[34] Yan YB, Li JM, Xiao E, An JG, Gan YH, Zhang Y (2014). A pilot trial on the molecular pathophysiology of traumatic temporomandibular joint bony ankylosis in a sheep model. Part II: The differential gene expression among fibrous ankylosis, bony ankylosis and condylar fracture. J Craniomaxillofac Surg.

[35] Zhang J, Sun X, Jia S, Jiang X, Deng T, Liu P, Hu K (2019). The role of lateral pterygoid muscle in the traumatic temporomandibular joint ankylosis: a gene chip based analysis. Mol Med Rep.

[36] Zhang J, Sun X, Jia S, Jiang X, Deng T, Liu P, Hu K (2019). The role of lateral pterygoid muscle in the traumatic temporomandibular joint ankylosis: a gene chip based analysis. Mol Med Rep.

[37] Yan YB, Zhang Y, Gan YH, An JG, Li JM, Xiao E (2013). Surgical induction of TMJ bony ankylosis in growing sheep and the role of injury severity of the glenoid fossa on the development of bony ankylosis. J Craniomaxillofac Surg.

[38] Yan YB, Liang SX, Shen J, Zhang JC, Zhang Y (2014). Current concepts in the pathogenesis of traumatic temporomandibular joint ankylosis. Head Face Med.

[39] Cheung LK, Shi XJ, Zheng LW (2007). Surgical induction of temporomandibular joint ankylosis: an animal model. J Oral Maxillofac Surg.

[40] Li Z, Zhang W, Li ZB (2009). Induction of traumatic temporomandibular joint ankylosis in growing rats: a preliminary experimental study. Dent Traumatol.

[41] Clark DP, Badea CT (2021). Advances in micro-CT imaging of small animals. Phys Med.

[42] Wu D, Yang XJ, Cheng P, Deng TG, Jiang X, Liu P, Liu CK, Meng FW, Hu KJ (2015). The lateral pterygoid muscle affects reconstruction of the condyle in the sagittal fracture healing process: a histological study. Int J Oral Maxillofac Surg.

[43] Nakatani T, Partridge NC (2017). MEF2C Interacts With c-FOS in PTH-Stimulated Mmp13 Gene Expression in Osteoblastic Cells. Endocrinology.

[44] Chen D, Kim DJ, Shen J, Zou Z, O'Keefe RJ (2020). Runx2 plays a central role in Osteoarthritis development. Journal of orthopaedic translation.

[45] Lehtola T, Nummenmaa E, Tuure L, Hämäläinen M, Nieminen RM, Moilanen T, Pemmari A, Moilanen E (2022). Dexamethasone Attenuates the Expression of MMP-13 in Chondrocytes through MKP-1. Int J Mol Sci.

[46] Takeuchi K, Ogawa H, Kuramitsu N, Akaike K, Goto A, Aoki H, Lassar A, Suehara Y, Hara A, Matsumoto K (2021). Colchicine protects against cartilage degeneration by inhibiting MMP13 expression via PLC-γ1 phosphorylation. Osteoarthritis Cartilage.

[47] Saiganesh S, Saathvika R, Arumugam B, Vishal M, Udhaya V, Ilangovan R, Selvamurugan N (2019). TGF-β1-stimulation of matrix metalloproteinase-13 expression by down-regulation of miR-203a-5p in rat osteoblasts. Int J Biol Macromol.

[48] Zhou ZB, Huang GX, Fu Q, Han B, Lu JJ, Chen AM, Zhu L (2019). circRNA.33186 Contributes to the Pathogenesis of Osteoarthritis by Sponging miR-127–5p. Mol Ther.

[49] Catheline SE, Hoak D, Chang M, Ketz JP, Hilton MJ, Zuscik MJ, Jonason JH (2019). Chondrocyte-Specific RUNX2 Overexpression Accelerates Post-traumatic Osteoarthritis Progression in Adult Mice. J Bone Miner Res.

[50] Yu H, Yao S, Zhou C, Fu F, Luo H, Du W, Jin H, Tong P, Chen D, Wu C (2021). Morroniside attenuates apoptosis and pyroptosis of chondrocytes and ameliorates osteoarthritic development by inhibiting NF-κB signaling. J Ethnopharmacol.

